# Application of Silver Sulfadiazine Cream With Early Surgical Intervention in Patients Suffering From Combined Burn-Blast Injury Facial Tattoos

**DOI:** 10.5812/traumamon.3935

**Published:** 2012-05-26

**Authors:** Ali Ebrahimi, Mohammad Hosein Kalantar Motamedi

**Affiliations:** 1 Trauma Research Center, Baqiyatallah University of Medical Sciences, Tehran, IR Iran

**Keywords:** Silver Sulfadiazine, Face, Burns, Blast Injuries

## Abstract

Severe combined burn-blast injury is a great challenge to surgical teams due to its high mortality. It also results in unsightly traumatic tattoos.

The aims of these case reports were to clarify the clinical characteristic of the dynamite explosion burn-blast facial injuries and discuss appropriate management of these patients. We report two patients suffering from facial burn-blast injury following dynamite explosion in which after primary stabilization, silver sulfadiazine cream was applied to the wounds and 12 hours later the wounds were cleaned under general anesthesia with vigorous saline solution irrigation and brushing. The foreign particles were meticulously removed from wounds and simultaneous repairing of defects was done with nylon 6-0 sutures. We conclude application of silver sulfadiazine cream on facial burn-blast injury tattoos several hours before surgical removal of particles is highly efficacious in facilitating particle removal and attaining a good result following surgical intervention, and primary repair. Treatment of combined burn-blast tattoos is different from other types of tattoos not associated with burns. Debridement and removal of foreign particles under general anesthesia from skin immediately and primary reconstruction of wounds is essential. We recommend application of the topical agent silver sulfadiazine to wounds about 12 hours before surgical intervention.

## 1. Background

Severe burn-blast injury is a great challenge to surgical teams due to its high mortality. Traumatic tattoos can result from injuries to the skin (1). Soldiers often develop tattoo as a result of explosions and may have explosive particles embedded in their skin. We present 2 cases of combined burn-blast tattoo management following dynamite explosion.

## 2. Objectives

The aims of these case reports were to clarify the clinical characteristic of the dynamite explosion burn-blast facial injuries and discuss the correct management of these patients.

## 3. Case Report

**Figure 1.**
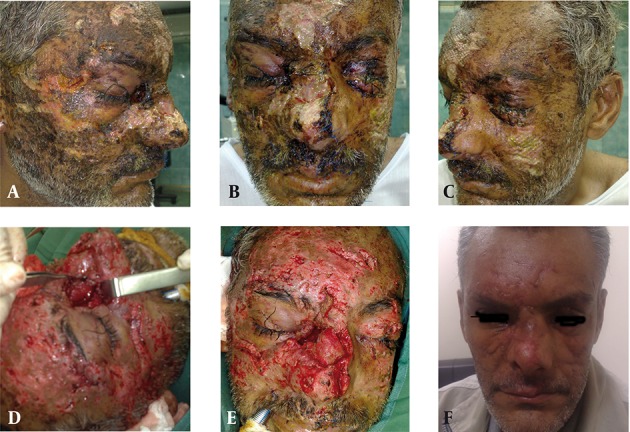
A 42 year-old man with burn-blast traumatic tattoo, a,b,c-before operation, d,e-intraoperative view, f-six months postoperation.

Case-1, is a 42 year- old man brought to hospital 48 hours after facial burn-blast injury due to dynamite explosion during road construction. He had traumatic tattoo and grade 2 facial burns (Figure 1). After primary stabilization of the patient, silver sulfadiazine cream was applied to facial burns with dressing for 12 hours before surgical intervention.

**Figure 2.**
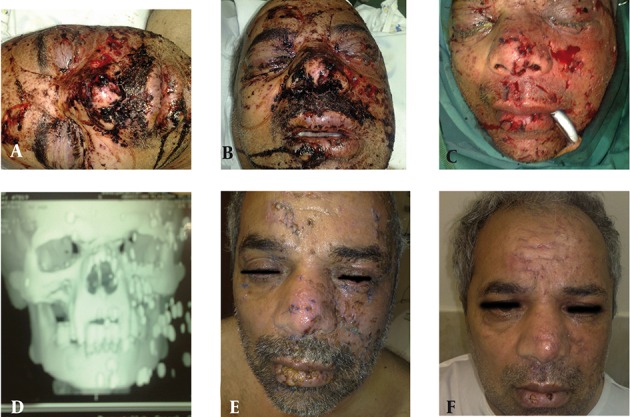
A 50 year-old man with burn-blast traumatic tattoo, a,b-before operation, c-intra operative view, d-x-ray view, e-early post operation, f-three months postoperation.

He had multiple deep lacerations on face with dynamite particles in subcutaneous tissues and the maxillary sinus; also he had blindness of left eye due to dynamite particles. In addition, he had diffuse burns of his face (Fig. 1). In the operating room, during surgery his wounds were explored and cleaned with vigorous saline solution irrigation and brushing and particles were meticulously removed from his wounds (Figure 1) and simultaneous repair of defects were done with nylon 6-0 sutures. Case-2, was a 50 year- old man brought to hospital 6 hours after facial burn-blast due to dynamite explosion in an accident. He had traumatic tattoos with dynamite particles and multiple glass particles in facial subcutaneous tissues and grade 2 patchy burns. After primary stabilization, we brought him to the operating room, and under general anesthesia his wounds were explored and cleaned with vigorous saline solution irrigation and brushing. Particles (glass and dynamite) were meticulously removed from his wounds (Figure 2), and simultaneous repairs of defects were done with nylon 6-0 sutures.

In both cases, after primary stabilization of the patient we applied silver sulfadiazine cream to the facial burns with dressings for 12 hours before surgical intervention.

## 4. Discussion

The first patient had facial burn and blindness of left eye due to dynamite particles; also he had defects of nasal dorsum and a 1.5 x 2 cm hole in the maxillary sinus from a nasal defect. A large dynamite particle was seen in the floor of the maxillary sinus. He also had diffuse dynamite particles in subcutaneous tissues of face. We applied silver sulfadiazine cream to the face before operation as this topical antimicrobial cream is widely used in burn patients. In the operation room vigorous washing and brushing of wounds were done and particles were removed by forceps. Simultaneous repair of wounds was then carried out after refreshing of edges and debridement. Defects of the nasal dorsum were reconstructed by mucosal turndown flaps and a simultaneous skin graft from the supraclavicle area. Because of different degrees of burns of facial skin, flap elevation from adjacent tissues was not possible. Six-month post operation evaluation photography (Figure 1) demonstrated acceptable results following early surgical intervention and removal of traumatic debris. In our second case a similar approach was applied and a 3-month post -operation photograp demonstrated an acceptable result. We conclude that the application of silver sulfadiazine cream on wounds 12 hours before surgical removal of particles is highly efficacious for aiding surgical intervention in such cases. Additionally, primary repair of facial defects is an ideal. We removed all sutures on the fifth day post operation. After tattoo removal, silicone sheets were put on suture lines for the prevention of scar formation. We performed similar management in all 6 of our cases with good results in long-term follow-ups.

There are many causes for skin tattoos, including trauma, explosions and cosmetic. Tattooing has been around since the early beginning of modern civilization (2). Traumatic tattooing is the embedding of a myriad of particles driven into the skin. The pigment granules will leave permanent dark blemishes if not removed immediately and can cause tattoo deformations of such magnitude that the patients future life is gravely affected (3). It is likely that tattoos have been removed for sometime using surgical excision, this method effectively removes the entire tattoo; however, it replaces it with a linear surgical scar (4-8). We removed dynamite particles under general anesthesia with surgical excision and linear closure of defects after washing and brushing of wounds, although some linear scars remained on the face (Figure 1 & 2), with regards to extension of burn-blast damage, it is a good choice for treatment of these patients. It is clear if we could remove tattoo particles in early post trauma, its complications may be minimized.

Burn-blast combined injury is caused by 2 injury factors: heat and blast, which damage the body at the same time or in sequence. The incidence of the combined injury is high either in wartime or in peace and the mortality is much higher than that of an injury due to a single injury factor (9). In the acute removal of burn-blast tattoo, there is no place for laser therapy because of different nature of this kind of tattoo, but it is a useful method for treatment of scars later on. With the advent of Q-switched lasers in the late 1960, outcomes of traumatic tattoo removal changed dramatically. In addition to their selective absorption by the pigment, the extremely short pulse duration of Q-switched lasers has made them the gold standard for tattoo removal (10). In burn-blast tattoos, foreign particles (dynamite particles) in skin necessitated that these particles be removed by surgical intervention immediately after injury for better aesthetic outcome. Bukhari reported tattoo in Arabian society used to have a cosmetic importance on the face of females, these were usually amateur tattoos done by non professional women in the tribes, nowadays Q-switched alexandrite laser is considered an effective method of tattoo removal (11).

For more effective treatment of facial burn-blast tattoos, we recommend application of topical silver sulfadiazine and early surgical removal of explosion debris and treatment of facial burns for better cosmetic results. Also it is safe to repair skin defects primarily in burn-blast cases after removing particles. 

## 5. Conclusions

The treatment of tattoos is a challenge for plastic surgeons and we need further studies in future. Application of silver sulfadiazine before surgical intervention helps to better remove embedded particles and decrease traumatic tatoos.
